# Effects of Physical Exercise on Circulating Serotonin Levels: A Systematic Review and Meta-Analysis

**DOI:** 10.3390/healthcare14040532

**Published:** 2026-02-21

**Authors:** Aarón Barrero-Osorio, Juan Manuel Franco-García, Damián Pereira-Payo, Miguel Rodal, Jorge Pérez-Gómez

**Affiliations:** 1 Health, Economy, Motricity and Education (HEME) Research Group, Faculty of Sport Sciences, University of Extremadura, 10003 Caceres, Spain; aaronbarreroosorio@gmail.com (A.B.-O.); jorgepg100@unex.es (J.P.-G.); 2 BioẼrgon Research Group, Faculty of Sport Sciences, University of Extremadura, 10003 Caceres, Spain; mrodal@unex.es

**Keywords:** 5-Hydroxytryptamine, modulation, training, physical activity

## Abstract

Serotonin is a neurotransmitter that plays a crucial role in mood regulation and has several health-related functions in humans. Physical exercise (PE) has been proposed as a potential non-pharmacological strategy to modulate serotonin levels. Objective: This systematic review and meta-analysis aimed to investigate the effect of PE on circulating serotonin concentrations. Methods: Following PRISMA guidelines, a systematic search was conducted in PubMed, Web of Science, and Scopus, identifying 938 records. Randomized controlled trials with a PE intervention group and a non-PE usual care control group that meet the inclusion criteria were selected. Data were synthesized using a random-effects meta-analysis with standardized mean differences (SMD), and sensitivity analyses were performed using alternative pre–post correlation assumptions (r = 0.6–0.9). Study quality was assessed using the PEDro scale. Results: Five randomized controlled trials comprising 116 participants, all of them females, were included. The pooled analysis showed a non-significant effect of PE on circulating serotonin levels (SMD 1.48; *p* = 0.135; 95% CI −0.46 to 3.42), with extremely high heterogeneity (I^2^ = 94.63%). Sensitivity analyses produced similar non-significant results and consistently high heterogeneity, indicating robustness of the original model. Methodological quality ranged from acceptable to good (PEDro scores 4–7). Conclusions: Current evidence, derived exclusively from female participants, does not support a significant effect of PE on serum or plasma serotonin levels. The substantial heterogeneity among studies and the generally moderate methodological quality limit the strength of the conclusions and generalizability of the conclusions. Further, high-quality randomized controlled trials with standardized measurement protocols are needed to clarify the influence of exercise on circulating serotonin levels.

## 1. Introduction

Over the past decades, research in neurobiology has increasingly focused on the role of neurotransmitters in regulating key physiological and psychological functions [1]. Among the wide range of neurotransmitters involved in human biology, serotonin has received particular attention due to its involvement in multiple regulatory processes across both central and peripheral systems. While serotonin is widely recognized for its role in affective regulation, it also contributes to a broad range of physiological functions relevant to overall health [2].

This neurotransmitter participates in multiple biological processes, including the regulation of mood, sleep, appetite, and motivation [3]. Altered serotonergic function has been consistently associated with various psychiatric disorders including depression, anxiety, and obsessive compulsive disorders [4]. These associations mainly reflect alterations in central serotonergic pathways and should not be directly extrapolated to peripheral serotonin dynamics. Beyond mental health, serotonin also plays a relevant role in multiple systemic physiological processes with implications for overall health.

Due to their high prevalence and chronic course, these conditions represent a major public health challenge, substantially impairing quality of life and generating substantial economic costs. In Europe, mental disorders account for nearly €600 billion annually, representing about 4% of GDP [5]. Accordingly, a considerable proportion of this economic burden is attributable to long-term pharmacological management, particularly the widespread use of selective serotonin reuptake inhibitors (SSRIs), which aim to increase serotonin availability within the central nervous system [6]. Although effective for many individuals, SSRIs are frequently associated with adverse effects that may limit adherence and long-term treatment success [7]. The substantial direct and indirect costs associated with these treatments further underscore the broader health and economic relevance of serotonergic regulation [8,9].

In humans, serotonin is synthesized both centrally in the brain and peripherally, predominantly in the gastrointestinal tract. Importantly, these two serotonin pools are functionally and anatomically distinct. Due to the presence of the blood–brain barrier, circulating serotonin does not cross into the central nervous system, and therefore peripheral serotonin cannot be considered a direct proxy for central serotonergic activity or brain neurotransmission [10]. While approximately 90–95% of total serotonin is produced peripherally, mainly by enterochromaffin cells in the gut, this serotonin primarily exerts local and systemic functions unrelated to synaptic neurotransmission, including roles in metabolic regulation, gastrointestinal motility, vascular tone, immune modulation, and platelet function [11,12].

In this context, physical exercise (PE) has emerged as a promising approach to modulate serotonin levels through non-pharmacological mechanisms [13]. Beyond its well-known general health benefits [14], exercise has been shown to influence neurotransmitter systems for decades [15]. Some evidence indicates that moderate-intensity aerobic exercise is associated with changes in serotonergic activity, including alterations detectable in peripheral circulation, although the extent to which these changes reflect central versus peripheral mechanisms remains uncertain [16,17,18]. In contrast, some strength training interventions have reported reductions in serotonin levels [19]. However, the current literature remains unclear regarding the comparative effects of aerobic versus strength training, as well as the influence of exercise characteristics such as intensity, duration, and frequency on serotonin modulation. Given these mixed findings and the heterogeneity of exercise protocols in the literature, including differences in intensity, duration, and exercise type, it is important to systematically analyze the existing studies to clarify how PE impacts serotonin.

Considering all the above, the objective of this systematic review and meta-analysis was to investigate the effect of PE on circulating serotonin levels, and to characterize exercise-related changes in peripheral serotonergic physiology.

## 2. Materials and Methods

This systematic review and meta-analysis were conducted in accordance with Preferred Reporting Items for Systematic Reviews and Meta-Analyses (PRISMA) guidelines. This review was not previously registered, and neither has a review protocol.

### 2.1. Search Strategy

The search was conducted in the PubMed, Scopus, and Web of Science (WOS) databases, covering from the year of the first publication (1983) to 4 February 2025. The Population, Intervention, Comparison, and Outcome (PICO) strategy was used [20] focusing on terms related to humans, adults, PE, randomized controlled trials (RCTs), and serotonin. The search string used was:

Pubmed: (serotonin AND (“physical activity” OR exercise OR “physical exercise”) AND (“randomized controlled trial” OR RCT) AND (human* OR adult*)).

Scopus: (TITLE-ABS-KEY (serotonin AND “physical activity” OR exercise OR “physical exercise” AND “randomized controlled trial” OR RCT) AND TITLE-ABS-KEY (human* OR adult*)).

WOS: (((serotonin) AND (“physical activity” OR exercise OR “physical exercise”)) AND ((“randomized controlled trial” OR RCT))) AND (human* OR adult*).

The references for the documents included were screened for potentially eligible studies.

### 2.2. Inclusion and Exclusions Criteria

The inclusion criteria for the studies were: (1) Participants: individuals over 18 years old, (2) Intervention: implementation of PE programs, (3) Outcomes: measurement of serotonin levels (4) Study type: Randomized Controlled Trials.

The exclusion criteria were: (1) Studies combining PE with other interventions, and (2) use of SSRIs or serotonergic drugs.

### 2.3. Study Selection

Two authors (A.B.-O. and J.M.F.-G.) conducted a selection process to examine the studies retrieved from the initial search. First, they reviewed the titles and abstracts using Zotero bibliographic manager to assess their suitability [21]. Then, the full texts of those that met the inclusion criteria were examined. The selected studies were compared and analyzed to ensure alignment with the objectives of this meta-analysis. In cases of doubt, decisions were discussed with a third author (J.P.-G.) and a decision was reached.

### 2.4. Methodological Quality Assessment

The methodological quality of the selected studies was assessed using the Physiotherapy Evidence Database (PEDro) scale [22]. The studies were categorized as excellent (9–10 points), good (6–8), acceptable (4–5), or low quality (≤3) [23].

### 2.5. Data Extraction and Analysis

Data extraction was carried out through a detailed review of the original articles to ensure accuracy. The primary data extracted from each study were the pre- and post-intervention means and standard deviations. Additional data extracted included exercise modality, intervention duration, weekly training frequency, exercise program intensity, completion rate, number of dropouts, reasons for dropout, outcome measures assessed, study design, and participants’ age, sex, height, weight, and baseline characteristics.

Serotonin concentrations were measured in serum in most studies, while one study assessed plasma serotonin levels [24]. It is important to note that serum and plasma serotonin concentrations may differ in absolute values due to platelet activation and serotonin release during the clotting process in serum samples. However, both matrices reflect peripheral circulating serotonin. The use of standardized mean differences (SMD) and the analysis of within-study pre–post changes reduce the impact of potential scale differences between biological matrices.

For meta-analysis, mean change scores were obtained by subtracting baseline mean values to post-intervention mean values. The standard deviation (SD) was calculated as described by Higgings et al. [25].

Jamovi (Jamovi 2.3.26) in its MAJOR module was used to conduct the meta-analysis. A randomized effect model with standardized mean differences (SMD) as effect size model measure was used. Heterogeneity among included studies was assessed using I^2^, Tau, Tau^2^, H^2^ and Q. SMD was used as the effect size because serotonin concentrations were measured using different assays and reported in different units across studies, requiring standardization.

Publication bias and small-study effects were explored using the Doi plot and the Luis Furuya–Kanamori (LFK) index. First, standardized mean differences (SMDs) and their corresponding standard errors (SE_SMD) were obtained for each study from the primary meta-analysis. These estimates were then imported into the R statistical environment (R version 4.3.3), where a random-effects meta-analysis model was fitted using the “meta” and “metasens” packages. The Doi plot and LFK index were subsequently generated in R following the approach proposed by Furuya-Kanamori et al. [26], using study-specific SMDs and their standard errors. This approach was chosen over traditional funnel plots due to its improved sensitivity in detecting asymmetry, particularly in meta-analyses with a small number of studies (≥5 and <10 studies). Given the limited number of included studies and the substantial between-study heterogeneity, all publication bias analyses were considered exploratory and interpreted with caution. Egger’s regression test was also conduced.

Sensitivity analyses were conducted by recalculating the meta-analysis using alternative assumptions for the pre–post correlation coefficient (r = 0.6, 0.7, 0.8, and 0.9), while keeping the other model specifications constant, with the purpose of assessing the robustness of the pooled estimates.

Exploratory meta-regression analyses were conducted using random-effects models with restricted maximum likelihood estimation to investigate potential sources of heterogeneity. Intervention duration and weekly training frequency were examined as continuous moderators. Meta-regressions were performed separately for each moderator. Given the small number of studies included, all findings were interpreted as exploratory.

## 3. Results

### 3.1. Study Design and Sample

**Figure 1:**
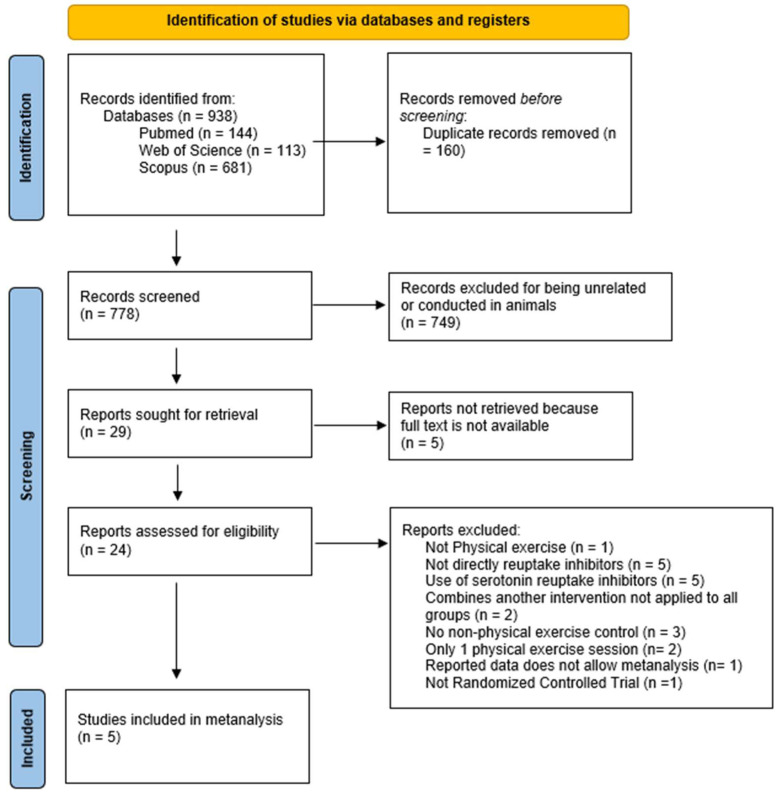
Flow diagram.

From an initial pool of 938 records, 24 were selected for full-text review, resulting in 5 studies being included in the final review (Figure 1). Five studies identified for full-text assessment could not be retrieved despite attempts to access the full manuscripts and were therefore classified as unretrieved reports [27,28,29,30,31]. Among the remaining studies, several were ultimately excluded for the following reasons: lack of a control group [32,33,34], an intervention duration consisting of only a single training session [35,36], and failure to follow a randomized controlled trial (RCT) design [37]. A further search for additional references did not yield any more records that met the inclusion criteria, so the final analysis comprised 5 studies.

The total sample size across these studies was 116 participants, with sample sizes per study ranging from 16 to 24. Participants’ characteristics are provided in Table 1 and details of the trials are provided in Table 2. All studies included in this review comprised female participants only, which should be considered when interpreting the generalizability of the findings.

### 3.2. Methodological Quality Assessment

Table 3 shows the scores obtained in the methodological quality assessment using the PEDro scale [22]. Two of the studies demonstrated good methodological quality [24,38], while the other three were rated as acceptable [19,39,40].

All studies, randomly allocated participants; however, only one performed concealed allocation [38]. Three studies ensured baseline comparability between groups [19,24,38]. None of the studies implemented participant or therapist blinding, although two blinded assessors [19,24]. Four studies conducted adequate follow-up [24,38,39,40]. All studies reported results for all participants. But only one did not compare groups with each other [38].

### 3.3. Dropouts and Reasons

A total of 11 dropouts were reported across two studies [19,40], as shown in Table 2. In the Y.-S. Kim et al., (2019) [19] trial, 4 participants dropped out due to relocation, hospitalization, and for personal reasons. For Rodziewicz-Flis et al. (2022) [40], 7 participants left due to lack of willingness, knee pain, low attendance, or reasons unrelated to the study.

### 3.4. General Characteristics of the Interventions

The main characteristics of the interventions are detailed in Table 4. The included studies consisted of randomized clinical trials, where participants were assigned to experimental and control groups, with the control group not being involved in any PE program.

In experimental groups, various forms of exercise were used, including aerobic training, strength training, balance, dance, Pilates and combat sports such as Taekwondo.

The duration of the interventions ranged from 8 to 24 weeks. The weekly training frequency ranged from 2 to 5 sessions. Intensity also varied among studies: 50–80% of percentage of maximum heart rate, and 9–13 rating of perceived exertion.

### 3.5. Main Results: Effects of Physical Exercise on Serotonin

**Figure 2:**
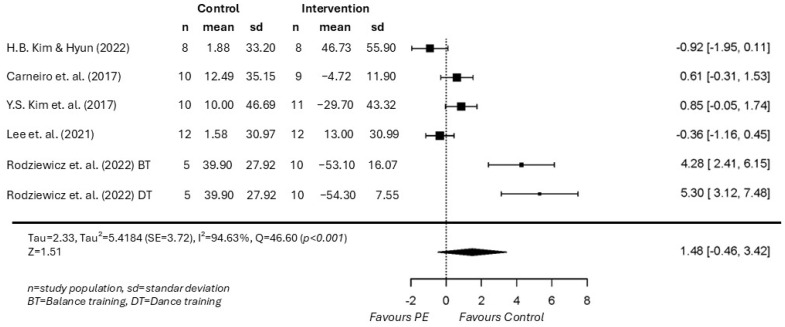
SMD between PE and control group for circulating Serotonin levels [19,24,38,39,40].

The overall results, derived from studies of acceptable to good methodological quality according to the PEDro scale, showed a non-significant effect of PE on serotonin levels (SMD 1.48; *p* = 0.135; 95% CI −0.46 to 3.42), with the direction of the effect favoring the control group. Very high heterogeneity was found among studies (I^2^ = 94.63%, Tau = 2.33, Tau^2^ = 5.4184 (SE = 3.7246) and H^2^ = 18.62). Consistent with this, the 95% prediction interval (−3.48 to 6.44) indicated that the true effect could range from negative to markedly positive across different settings. The analysis of the residuals did not identify any study as an outlier. Additionally, the Egger’s regression test was significant, this evidence should be interpreted with caution due to the small number of studies included and the very high level of heterogeneity observed. In this context, a significant Egger test result does not necessarily indicate the presence of publication bias (Figure 2).

### 3.6. Sensitivity Analysis

The sensitivity analysis was performed to assess the impact of alternative assumptions for the pre–post correlation (r = 0.6, 0.7, 0.8, and 0.9) on the pooled estimates. The results showed that varying the correlation coefficient produced only modest numerical changes in the effect size. All resulting models yielded non-significant pooled effects with wide confidence intervals overlapping zero, and these differences were insufficient to alter the statistical interpretation of the findings. Across all sensitivity scenarios, heterogeneity measurements remained extremely high (I^2^ > 95%), indicating that the substantial between-study variation persisted regardless of the assumed correlation (Table 5). The consistency of the results across a wide range of assumed pre–post correlations suggests that the main conclusions are not driven by the specific choice of correlation coefficient but rather reflect the underlying variability and uncertainty of the available evidence.

### 3.7. Publication Bias

**Figure 3:**
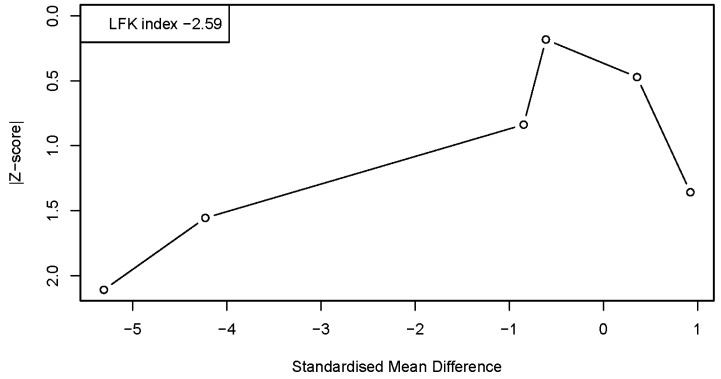
DOI plot and LFK index for the meta-analysis model.

Publication bias and small-study effects were explored using the Doi plot in combination with the Luis Furuya–Kanamori (LFK) index. In the primary analysis including all available intervention arms (k = 6 effect sizes derived from 5 studies), the Doi plot showed marked asymmetry, with an LFK index of −2.59, indicating major asymmetry (Figure 3). The direction of the asymmetry suggested an overrepresentation of studies reporting relatively large negative standardized mean differences. Diagnostics based on standardized residuals did not identify any study as a formal statistical outlier; however, influence analyses indicated that the DT intervention arm of. [40] exerted a relatively high influence on the pooled estimate. Overall, the observed asymmetry is likely related to the small number of included studies, substantial between-study heterogeneity, and the presence of highly influential effect sizes rather than to selective reporting.

**Figure 4:**
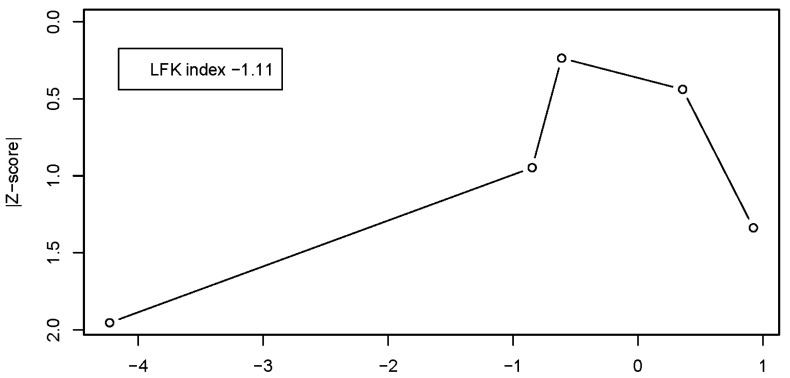
DOI plot and LFK index for the reduced meta-analysis model (k = 5).

As a sensitivity analysis, the Doi plot was re-estimated after excluding the DT intervention arm of [40]. In this reduced model (k = 5), the Doi plot appeared notably more symmetrical and the LFK index decreased to −1.11, indicating only minor asymmetry (Figure 4). This attenuation suggests that the marked asymmetry observed in the primary analysis was largely driven by the influence of this single study rather than reflecting consistent small-study effects.

Consistent with these findings, an exploratory sensitivity meta-analysis was conducted excluding the same DT intervention arm of [40]) (see Table 6). In this reduced model (k = 5), the pooled standardized mean difference was attenuated (SMD = 0.77, 95% CI −0.83 to 2.37), with the confidence interval including the null value, while substantial heterogeneity persisted (I^2^ = 92.0%; Q = 27.21, *p* < 0.001). These results indicate that the magnitude of the pooled effect estimate was sensitive to the inclusion of this influential study, whereas the overall pattern of high between-study heterogeneity remained unchanged.

### 3.8. Exploratory Meta-Regression

Due to the low number of studies eligible for this meta-analysis (*n* < 10), the results of the meta-regression can only be considered as an exploratory analysis. Thus, the meta regression did not identify any significant moderators of the effect size. Regarding duration of the intervention, the moderator coefficient was small and non-significant (Estimate = −0.06; *p* = 0.773; 95% CI −0.50 to 0.37), and the meta-regression did not reveal a detectable association between intervention duration and effect size. Residual heterogeneity remained extremely high (I^2^ = 95.51%; τ^2^ = 6.98), and the model explained none of the between-study variance (R^2^ = 0%). Similarly, weekly training frequency was not a significant predictor of the effect size (Estimate = −0.28; *p* = 0.82; 95% CI −2.67 to 2.12). The heterogeneity estimates in this model were nearly unchanged from the unmoderated analysis (I^2^ = 95.15%; Tau^2^ = 7.03), and the proportion of variance explained was also zero (R^2^ = 0%) (Table 7).

It should also be noted that participant age and sex were not examined as potential moderators. Age could not be explored because one of the included studies did not report this information, and sex was not considered a moderating variable because all studies exclusively included female participants.

## 4. Discussion

This systematic review and meta-analysis aimed to investigate the effect of PE on circulating serotonin levels. Five randomized controlled trials were included, with methodological quality ranging from acceptable to good. The pooled results did not show a significant effect of PE on circulating serotonin, and the confidence intervals spanned both negative and positive values, indicating substantial uncertainty regarding the true direction and magnitude of the effect. High between-study heterogeneity and a wide prediction interval were found, suggesting that true effects may vary across different contexts. Thus, in the presence of substantial heterogeneity, the pooled estimate should be interpreted as a summary measure rather than a precise representation of a common underlying effect. Sensitivity analyses further supported the robustness of the main findings, as alternative pre–post correlation assumptions (r = 0.6–0.9) yielded similarly non-significant results with high heterogeneity as well. In addition, exploratory assessments of small-study effects using Doi plots suggested that the observed asymmetry was sensitive to the influence of individual studies, further supporting a cautious interpretation of the pooled estimates. Finally, exploratory meta-regression analyses did not identify any significant moderators, with neither intervention duration nor weekly training frequency explaining the observed variability between studies, however, these results should be interpreted with caution due to the limited number of included studies and the exploratory nature of the analyses.

Although the overall pooled effect was non-significant, the individual studies showed marked variability in both the direction and magnitude of changes in circulating serotonin levels, underscoring the heterogeneity observed across trials. Specifically, Rodziewicz-Flis et al. (2022) and Y.-S. Kim et al. (2019) [19,40] reported significant decreases in serotonin levels following dance-based and strength training interventions, respectively, both conducted in older women populations, whereas Carneiro et al. (2017) [39] observed a similar downward trend after aerobic exercise that did not reach statistical significance. In contrast, H.-B. Kim and Hyun (2022) [38], in a sample of pregnant women undergoing an online Pilates program, reported a statistically significant increase in serotonin levels, while Lee et al. (2021) [24] found a significant group-by-time interaction favoring increased serotonin levels following a 16-week taekwondo intervention in obese postmenopausal women. These divergent findings across populations, exercise modalities, and intervention characteristics suggest that the effect of physical exercise on circulating serotonin levels may be context-dependent, which likely contributes to the substantial heterogeneity observed and support a cautious interpretation of the pooled effect estimate.

Evidence from acute exercise studies suggests that serotonin responses may differ from those observed after longer-term interventions. For instance, Tsai and Pan et al. (2023) [36], reported a significant increase in serotonin levels after a session of moderate-intensity exercise. Also, in a single session of aerobic exercise, Zimmer et al., (2016) [35] found a significant increase in circulating serotonin levels regardless of PE intensity. These acute responses are likely driven by transient neurochemical mechanisms, such as increased neuronal firing, substrate availability, or stress-related hormonal responses, which may differ from the regulatory and homeostatic adaptations induced by repeated training over time. This suggests that acute exercise-induced increases in serotonin do not necessarily translate into sustained long-term adaptations, which may partially explain the inconsistent findings observed across chronic exercise interventions. Importantly, the distinction between acute exercise responses and long-term training adaptations should be considered when interpreting these findings, as pooling both effects under a single physiological framework may oversimplify the complex neurobiological responses of serotonin to PE.

The combined effect of PE with selective serotonin reuptake inhibitors has been also studied, finding greater increases in serotonin levels for the group performing PE as co-therapy compared to the controls [41]. This suggests that exercise may enhance the effects of pharmacological treatments in mood regulation and mental health disorders [42].

Regarding PE type, it remains unclear whether different exercise induce distinct responses on serotonin levels. Although some studies have reported increases following aerobic or mind–body interventions [24,38,43] and decreases after strength or balance-based training [40], these findings should be interpreted as preliminary and exploratory rather than indicative of consistent modality-specific trends, given the very limited number of studies available for each exercise type and the substantial heterogeneity in intervention characteristics. Therefore, current evidence is insufficient to draw firm conclusions regarding differential effects of exercise modality on serotonin regulation. Future studies with adequately powered samples and standardized exercise protocols are needed to clarify whether true modality-specific effects on serotonin exist.

Finally, emerging evidence suggests that the effects of PE on serotonin may be mediated by interactions with other neurotransmitters, such as dopamine and GABA, which together may contribute to improved neurochemical balance and stress regulation [44]. The combined action of these neurotransmitter systems warrants further investigation and may provide a more comprehensive understanding of the neurobiological effects of exercise.

### Limitations

This meta-analysis has some limitations that should be acknowledged. Firstly, the studies included present substantial heterogeneity in terms of exercise protocols varying in type, intensity, duration, and frequency, which makes it difficult to draw firm and generalizable conclusions. An important limitation of the present evidence is that all included studies were conducted exclusively in female participants, which limits the generalizability of the findings to male population. Additionally, heterogeneity in age and health status across study populations, despite all participants being females, may have contributed to the lack of homogeneity in the observed results. Taken together, the exclusive inclusion of women combined with variability in age and baseline health status further constrains the external validity of the findings, limiting their applicability to other demographic groups, clinical populations, or age ranges. Furthermore, many studies had small sample sizes, limiting the statistical power and external validity of the findings. The limited number of studies included in the meta-analysis also reduces its statistical power, meaning that the results should be interpreted as exploratory rather than confirmatory. Additionally, one included study measured plasma serotonin whereas the others assessed serum levels. Although both matrices reflect peripheral serotonin physiology, differences in sample processing and platelet activation may introduce biological variability, potentially contributing to heterogeneity. Finally, while the methodological quality of the randomized controlled trials included was evaluated using the PEDro scale, the overall certainty of the evidence was not formally graded using the GRADE approach. Therefore, the strength of the conclusions should be interpreted with caution when translating these findings into clinical or public health recommendations. The generally low levels of blinding and allocation concealment across the included trials may have introduced performance and detection bias, which could have led to an overestimation of the pooled effect sizes.

## 5. Conclusions

This systematic review and meta-analysis found no conclusive evidence supporting a significant effect of PE on circulating serotonin levels. The wide confidence intervals and the extremely high between-study heterogeneity indicate substantial uncertainty and suggest that the true effects may vary considerably across populations and intervention contexts.

Importantly, circulating serotonin reflects peripheral serotonergic physiology and should not be interpreted as a direct marker of central serotonergic activity or mental health related mechanisms.

The pronounced heterogeneity observed across studies may be partly explained by differences in intervention and population characteristics, although the limited number of available studies precludes formal subgroup analyses. Further well-designed randomized controlled trials with larger sample sizes, standardized exercise protocols, and comprehensive reporting of participant characteristics are needed to clarify the conditions under which PE may influence circulating serotonin levels.

## Figures and Tables

**Table 1:** Participant characteristics and study design.

Study	Study Design	Sample Initial/Final	Group	n	Age (Years) Mean (SD) or Range	Height (cm) Mean (SD)	Weight (kg) Mean (SD)
(H.-B. Kim & Hyun, 2022a) [38]	RCT	Pregnant women (24–28 weeks) 16/16	CG	8	38.14 ± 1.39	163.82 ± 3.71	64.55 ± 2.52
			PG	8	39.71 ± 2.01	164.81 ± 4.43	62.71 ± 4.00
(Carneiro et al., 2017) [39]	RCT	Sedentary women 19/19	CG	10	18–65	-	-
			EG	9		-	-
(Y.-S. Kim et al., 2019) [19]	RCT	Older women with depression 25/21	CG	10	76.40 ± 3.27	152.77 ± 5.63	52.35 ± 2.86
			STG	11	76.10 ± 3.85	151.14 ± 5.42	54.74 ± 6.73
(Lee et al., 2021) [24]	RCT	Obese postmenopausal women 24/24	CG	12	57.5 ± 2.9	156.6 ± 3.3	62.6 ± 6.2
			EG	12	56.0 ± 2.9	157.4 ± 4.7	64.0 ± 5.8
(Rodziewicz-Flis et al., 2022) [40]	RCT	Older women 37/30	CG	10	73.4 ± 5.0	160 ± 4	70.5 ± 10.2
			BTG	10	74.3 ± 4.6	160 ± 4	65.0 ± 8.0
			DTG	10	72.1 ± 4.1	159 ± 3	71.2 ± 5.0

-: Not reported, SD: standard deviation, CG: Control Group, PG: Pilates Group, EG: Experimental Group, STG: Strength Training Group, BTG: Balance Training Group, DTG: Dance Training Group.

**Table 2:** Study design and characteristics.

Study	Study Design	Measurements	Sample Type	Completion Rate		
					*n*	Reasons
(H.-B. Kim & Hyun, 2022a) [38]	RCT	Body composition test, blood analysis, EPDS, PSS, and PSQI	Serum	100%		-
				100%	-	-
(Carneiro et al., 2017) [39]	RCT	Blood analysis pre/post intervention: dopamine, norepinephrine, epinephrine, serotonin, and cortisol	Serum	100%	-	-
				100%	-	-
(Y.-S. Kim et al., 2019) [19]	RCT	Blood analysis (serotonin, dopamine, epinephrine, norepinephrine) and SGDS-K	Serum	76.92%	3	Relocation, hospitalization, personal reasons
				91.67%	1	
(Lee et al., 2021) [24]	RCT	Body composition, serum lipid profiles, plasma serotonin and dopamine, cerebral blood flow velocity, PANAS, and SWLS	Plasma	100%	-	-
				100%		
(Rodziewicz-Flis et al., 2022) [40]	RCT	TUG, 6MWT, Determination Test, and blood levels of APP and serotonin	Serum	83.33%	2	Lack of motivation, knee pain, <80% attendance, unrelated reasons
				83.33%	2	
				76.92%	3	

-: Not reported, RCT: Randomized Controlled Trial, *n*: number of Dropouts.

**Table 3:** Results of the methodological quality assessment of the included studies.

Study	PEDro Scale Items											Score	Quality
	1	2	3	4	5	6	7	8	9	10	11		
(Carneiro et al., 2017) [39]	1	1	0	0	0	0	0	1	1	1	1	5	Acceptable
(H.-B. Kim & Hyun, 2022a) [38]	1	1	1	1	0	0	1	1	1	0	1	7	Good
(Lee et al., 2021) [24]	1	1	0	1	0	0	1	1	1	1	1	7	Good
(Rodziewicz-Flis et al., 2022) [40]	1	1	0	0	0	0	0	1	1	1	1	5	Acceptable
(Y.-S. Kim et al., 2019) [19]	1	1	0	1	0	0	0	0	1	0	1	4	Acceptable

PEDro Scale Items: 1: Eligibility criteria, 2: Random allocation, 3: Concealed allocation, 4: Baseline comparability, 5: Participants blinding, 6: Therapist blinding, 7: Assessor blinding, 8: Adequate follow-up, 9: Intention-to-treat analysis, 10: Between-group comparisons, 11: Point estimates and variability.

**Table 4:** Main characteristics of the interventions.

Study	Study Design	Group	Intervention			
			Exercise Type	Duration (Weeks)	Frequency (per Week)	Intensity
(H.-B. Kim & Hyun, 2022a) [38]	Pilot RCT	CG	None	8	-	-
		PG	Online Pilates program		2	50–60% HRmax RPE 11–13
(Carneiro et al., 2017) [39]	RCT	CG	No exercise	16	-	-
		EG	Aerobic exercise		3	72% HRmax RPE 12–13
(Y.-S. Kim et al., 2019) [19]	RCT	CG	None	24	-	-
		STG	Strength training program		3	RPE 9–13
(Lee et al., 2021) [24]	RCT	CG	No lifestyle change	16	-	-
		EG	Taekwondo sessions		5	50–80% HRmax
(Rodziewicz-Flis et al., 2022) [40]	RCT	CG	No lifestyle change	12	-	-
		BTG	Balance training		3	60–80% HRmax
		DTG	Dance training		3	60–80% HRmax

-: Not reported, RCT: Randomized Controlled Trial, Pilot RCT: Pilot Randomized Controlled Trial, HRmax: Maximum Heart Rate, RPE: Rating of Perceived Exertion (Borg Scale), BTG: Balance Training Group, CG: Control Group, DTG: Dance Training Group, EG: Experimental Group, PG: Pilates Group, STG: Strength Training Group.

**Table 5:** Sensitivity analysis shows the characteristics of the meta-analysis models for each explored pre-post correlation coefficients.

r	SMD	se	Z	*p*	CI Lower	CI Upper	Heterogeneity					
							Tau	Tau^2^	I^2^	H^2^	Q	*p*’
0.6	1.60	1.07	1.49	0.14	−0.50	3.70	2.52	6.38 (SE = 4.36)	95.29	21.24	50.19	<0.001
0.7	1.76	1.18	1.49	0.14	−0.55	4.06	2.78	7.73 (SE = 5.25)	95.96	24.76	54.85	<0.001
0.8	1.97	1.32	1.49	0.14	−0.62	4.56	3.13	9.80 (SE = 6.61)	96.64	29.748	61.51	<0.001
0.9	2.29	1.54	1.49	0.14	−0.73	5.31	3.66	13.42 (SE = 9.28)	97.31	37.13	73.33	<0.001

r = pre-post correlation coefficient used in the model, SMD = standardized mean difference, se = standard error, Z = Z-statistic, *p* = *p*-value of Z-statistic, CI = confidence interval, Tau and Tau^2^ = between-study heterogeneity, I^2^ and H^2^ = magnitude of heterogeneity, Q = Cochran’s Q test, *p*’ = *p*-value of Cochran’s Q test.

**Table 6:** Exploratory reduced meta-analysis model.

SMD	se	Z	*p*	CI Lower	CI Upper	Heterogeneity					
						Tau	Tau^2^	I^2^	H^2^	Q	*p*’
0.77	0.82	0.94	0.35	−0.83	2.37	1.74	3.0107 (SE = 2.3647)	92.01	12.52	27.21	<0.001

SMD = standardized mean difference, se = standard error, Z = Z-statistic, *p* = *p*-value of Z-statistic, CI = confidence interval, Tau and Tau^2^ = between-study heterogeneity, I^2^ and H^2^ = magnitude of heterogeneity, Q = Cochran’s Q test, *p*’ = *p*-value of Cochran’s Q test.

**Table 7:** Exploratory meta-regression models with Duration of intervention and Frequency of training as mediators.

		Estimate	se	Z	*p*	CI Lower	CI Upper
Duration of intervention	Intercept	2.45	3.45	0.71	0.478	−4.31	9.21
	Moderator	−0.06	0.22	−0.29	0.773	−0.50	0.37
Weekly frequency of training	Intercept	2.39	4.05	0.59	0.554	−5.537	10.32
	Moderator	−0.28	1.22	−0.23	0.820	−2.677	2.121

se = standard error, Z = Z-statistic, *p* = *p*-value of Z-statistic.

## Data Availability

No new data were created or analyzed in this study.

## Abbreviations

The following abbreviations are used in this manuscript:

6MWT	6-Minute Walk Test
APP	Amyloid Precursor Protein
BTG	Balance Training Group
CG	Control Group
CI	Confidence Interval
DTG	Dance Training Group
EG	Experimental Group
EPDS	Edinburgh Postnatal Depression Scale
H^2^	H-squared. Magnitude of Heterogeneity
HRmax	Maximum Heart Rate
I^2^	I-squared. Magnitude of Heterogeneity
PANAS	Positive And Negative Affect Schedule
PE	Physical Exercise
PEDro	Physiotherapy Evidence Database
PG	Pilates Group
PICO	Population, Intervention, Comparison, and Outcome
PRISMA	Preferred Reporting Items for Systematic Reviews and Meta-Analyses
PSQI	Pittsburgh Sleep Quality Index
PSS	Perceived Stress Scale
Q	Conchran’s Q Test
*p*’	*p*-value of Cochran’s Q test
*p*	*p*-value of Z-statistic
RCTs	Randomized Controlled Trials
RPE	Rating of Perceived Exertion
r	pre-post correlation coefficient used in the model
SD	Standard Deviation
SE	Standard Error
SGDS-K	Korean version of the Short-Form Geriatric Depression Scale
SMD	Standardized Mean Differences
SSRIs	Selective Serotonin Reuptake Inhibitors
STG	Strength Training Group
SWLS	Satisfaction With Life Scale
Tau and Tau^2^	Between-study heterogeneity
TUG	Timed Up and Go Test
WOS	Web Of Science
Z	Z-statistic
